# Loss of ubiquitin-specific peptidase 18 destabilizes 14-3-3ζ protein and represses lung cancer metastasis

**DOI:** 10.1080/15384047.2022.2054242

**Published:** 2022-04-07

**Authors:** Zibo Chen, Lin Zheng, Yulong Chen, Xiuxia Liu, Masanori Kawakami, Lisa Maria Mustachio, Jason Roszik, Katherine V. Ferry-Galow, Ralph E. Parchment, Xin Liu, Thorkell Andresson, Gerard Duncan, Jonathan M. Kurie, Jaime Rodriguez-Canales, Xi Liu, Ethan Dmitrovsky

**Affiliations:** a Thoracic/Head and Neck Medical Oncology, The University of Texas MD Anderson Cancer Center, Houston, TX, USA; b Cancer Research Technology Program, Frederick National Laboratory for Cancer Research, Frederick, MD, USA; c Melanoma Medical Oncology, The University of Texas MD Anderson Cancer Center, Houston, TX, USA; d Genomic Medicine, The University of Texas MD Anderson Cancer Center, Houston, TX, USA; e Clinical Pharmacodynamic Program, Applied/Developmental Research Directorate, Frederick National Laboratory for Cancer Research, Frederick, MD, USA; f Oncology and Translational Medicine, AstraZeneca, Gaithersburg, MD, USA; g Cancer Biology, The University of Texas MD Anderson Cancer Center, Houston, TX, USA

**Keywords:** Deubiquitinase, USP18, 14-3-3ζ, lung cancer and metastasis

## Abstract

Cancer metastasis is a major cause of cancer-related mortality. Strategies to reduce metastases are needed especially in lung cancer, the most common cause of cancer mortality. We previously reported increased ubiquitin-specific peptidase 18 (USP18) expression in lung and other cancers. Engineered reduction of USP18 expression repressed lung cancer growth and promoted apoptosis. This deubiquitinase (DUB) stabilized targeted proteins by removing the complex interferon-stimulated gene 15 (ISG15). This study explores if the loss of USP18 reduced lung cancer metastasis. USP18 knock-down in lung cancer cells was independently achieved using small hairpin RNAs (shRNAs) and small interfering RNAs (siRNAs). USP18 knock-down reduced lung cancer growth, wound-healing, migration, and invasion versus controls (P < .001) and markedly decreased murine lung cancer metastases (P < .001). Reverse Phase Protein Arrays (RPPAs) in shRNA knock-down lung cancer cells showed that 14-3-3ζ protein was regulated by loss of USP18. ISG15 complexed with 14-3-3ζ protein reducing its stability. Survival in lung adenocarcinomas (P < .0015) and other cancers was linked to elevated 14-3-3ζ expression as assessed by The Cancer Genome Atlas (TCGA). The findings were confirmed and extended using 14-3-3ζ immunohistochemical assays of human lung cancer arrays and syngeneic murine lung cancer metastasis models. A direct 14-3-3ζ role in controlling lung cancer metastasis came from engineered 14-3-3ζ knock-down in lung cancer cell lines and 14-3-3ζ rescue experiments that reversed migration and invasion inhibition. Findings presented here revealed that USP18 controlled metastasis by regulating 14-3-3ζ expression. These data provide a strong rationale for developing a USP18 inhibitor to combat metastases.

## Introduction

Lung cancer is the leading cause of cancer mortality for men and women.^1^ Despite advances in surgery, radiation therapy, chemotherapy, and immunotherapy survival outcomes for lung cancer patients need improvement.^1–4^ Lung cancers present as non-small cell lung cancer (NSCLC) or small cell lung cancer (SCLC); lung cancer five-year survival is only 17%.^1,2^ NSCLCs are often diagnosed with advanced stage when local treatments are rarely curative.^1,2^

One reason is the onset of metastasis that shortens survival and is an unmet medical need.^1,5,6^ Added challenges come when targeted therapies elicit resistance.^2,5^ Innovative ways to combat lung cancer are needed, especially when metastases occur. One approach is through a fuller understanding of lung cancer metastasis that would uncover new ways to intervene and improve clinical outcomes.

Ubiquitination alters the stability of growth regulatory proteins and affects cancer biology.^7–9^ This pathway contributes to diverse processes like cellular survival, growth, differentiation, and metastases.^7–9^ Ubiquitination engages three steps.^7–9^ The first is activation of ubiquitin by the ubiquitin-activating enzyme (E1). Ubiquitin is next transferred to the ubiquitin conjugating enzyme (E2). Ubiquitin ligase (E3) engages the last step with a ubiquitin-loaded E2 before a modified protein is degraded through the proteasome.^7–9^

Modification with interferon stimulated gene 15 (ISG15) is also activated by a three-step enzymatic cascade. This engages a distinct E1-activating enzyme (UBE1L), an E2-conjugating enzyme (UBCH8), and an E3 ligase (HERC5A) that complexes ISG15 to substrates.^9–11^ Unlike the proteasome-ubiquitination pathway, consequences of ISG15ylation are less well understood partly because fewer ISG15 complexed proteins are known and functionally validated.^9–11^

Deubiquitinases (DUBs) are a superfamily of enzymes that remove ubiquitin or ubiquitin-like species from substrate proteins.^12^ Of the more than 100 DUBs known ubiquitin-specific proteases (USPs) are a distinct family.^9,10,13^ The ubiquitin-specific peptidase 18 (USP18) DUB preferentially removes ISG15 from target proteins and by this opposes ISG15ylation effects.^9,10^ Enhanced USP18 expression occurs in lung and other cancers including those in the colon, rectum, brain, pancreas, cervix, and elsewhere.^9,14–20^ Our prior work reported that increased USP18 expression promoted tumorigenesis by stabilizing ISG15-conjugated proteins like PML/RARα, cyclin D1, and KRAS that affect carcinogenesis.^21–23^

The role of USP18 in metastasis needs to be elucidated. Addressing this gap in knowledge is the subject of this study. Consistent with a USP18 role in regulating metastasis is that USP18 alters the epithelial–mesenchymal transition (EMT).^24^ We sought to confirm and extend that prior work by learning if regulated USP18 expression alters lung cancer metastasis. Reverse Phase Protein Arrays (RPPAs) were performed to find proteins that were regulated by USP18 knock-down and could reduce lung cancer migration, invasion, and metastasis.

USP18 knock-down highlighted 14-3-3ζ protein. 14-3-3ζ is part of an acidic protein mammalian family with seven (β, γ, ε, η σ, θ, and ζ) isoforms.^25^ These interact with a network of proteins that influence cell cycle control, proliferation, migration, invasion, and other processes.^25^ Of these isoforms, 14-3-3ζ is abundantly expressed in different cancers including those in the lung, liver, breast, and elsewhere.^26–29^ Altered lung cancer proliferation, migration and invasion, and tumorigenesis were linked to changes in 14-3-3ζ expression.^26–29^

These findings implicated but did not confirm 14-3-3ζ as a critical anti-neoplastic target.^29–35^ Consequences of altered 14-3-3ζ expression need to be discerned. The findings presented here establish a critical role for ISG15 complex formation with 14-3-3ζ protein that alters 14-3-3ζ protein stability, lung cancer metastases in murine and human lung cancer survival.

Our findings established that USP18 inhibition reduced lung cancer metastasis by repressing 14-3-3ζ protein expression. A mechanistic link for this repression was shown through the complex formation between ISG15 and 14-3-3ζ proteins. Intriguingly, USP18 knock-down reduced lung cancer metastasis. RPPAs performed after USP18 knock-down highlighted 14-3-3ζ as a key mediator of the lung cancer metastasis process. Decreased 14-3-3ζ protein markedly reduced invasion and migration of lung cancer cells. In contrast, gain of 14-3-3ζ expression rescued this ability of lung cancer cells despite engineered USP18 repression.

Two distinct syngeneic murine lung cancer metastasis models were interrogated after transplantation of lung cancer cells having engineered USP18 repression as compared to controls. Added evidence came from interrogating 14-3-3ζ immunohistochemical expression profiles in primary versus metastatic lung cancers after USP18 knock-down versus controls. The findings were confirmed and extended by analysis of 14-3-3ζ profiles in TCGA and 14-3-3ζ immunohistochemical analyses of human lung cancer arrays as compared to the adjacent benign lung. Taken together, these data implicate USP18 as a therapeutic target for reducing lung cancer metastasis.

## Materials and methods

### Cell culture

Human (A549 and H1299) lung cancer, murine (344SQ, KC2, and 393P) lung cancer and 293T cell lines were cultured in RPMI-1640 medium (Thermo Fisher Scientific, Waltham, MA) supplemented with 10% fetal bovine serum (FBS) (General Electric, Boston, MA). Cells were cultured at 37°C in a humidified incubator with 5% CO_2_. A549, H1299 and 293T cell lines came from the American Type Culture Collection (ATCC) except for KC2, 344SQ, and 393P lung cancer cell lines that Dr. Jonathan M. Kurie (MD Anderson Cancer Center, Houston, TX) provided. All cell lines were authenticated, as described^36,37^ or by ATCC. Mycoplasma was assessed by the MycoAlert Mycoplasma Detection Kit (Lonza, Basel, Switzerland).

### Plasmids, shRNA, siRNAs, and transfection

Plasmids pCMV-HA-ISG15, pSG5-UBE1L, pCMV2-UBCH8 were described.^21,22^ Plasmids pCAGGS-6His-mISG15 (Addgene, Watertown, MA), pcDNA3-flag-HA-14-3-3ζ (Addgene, Watertown, MA), pCMV-eGFP-vector (Genecopiea, Rockville, MD) and pcDNA3-HA-14-3-3ζ (Addgene, Watertown, MA) were purchased. The TRC pLKO.1 lentiviral shRNAs repressing USP18 were purchased as were human USP18 shRNA: RHS4430-200230552 and RHS4430-200230647; murine USP18 shRNA: RMM4431-200398828 and RMM4431-200406549; control shRNA: RHS4349 (Dharmacon, Lafayette, CO). Independent transient transfection of logarithmically growing A549, H1299, KC2 and 344SQ lung cancer cells was by Lipofectamine 3000 Transfection Reagent (Thermo Fisher Scientific, Waltham, MA). EGFP-expressing plasmids assessed transfection efficiencies. Transfections were in triplicate and with at least three independent replicate experiments.

Independent transient transfection of A549, H1299, and 344SQ lung cancer cells was by small interfering RNAs (siRNAs) and the manufacturer’s methods for siRNA-mediated transfection experiments (Dharmacon, Lafayette, CO). Independent siRNAs to target USP18 or 14-3-3ζ and inactive control siRNAs were synthesized (Dharmacon, Lafayette, CO); siRNAs that targeted USP18 were as follows: USP18 human siRNA3

(5’-GGACUACCCUCAUGGCCUG-3’); USP18 human siRNA4

(5’-GCAAAUCUGUCAGUCCAUC-3’); USP18 murine siRNA1

(5’-CGUUGUUUGUCCAGCACGA-3’) and USP18 murine siRNA4

(5’-GAUCAUCGGUUCAUGGGAU-3’). Different siRNAs targeting 14-3-3ζ were: 14-3-3ζ human siRNA2 (5’-GCAGAUGGCUCGAGAAUAC-3’); 14-3-3ζ human siRNA3

(5’-GCCCGUAGGUCAUCUUGGA-3’); 14-3-3ζ murine siRNA1

(5’-GCUCGAGAAUACAGAGAGA-3’) and 14-3-3ζ murine siRNA3

(5’- GCAACGAUGUACUGUCUCU −3’). Transfection efficiency was by co-transfecting the siGLO Red Transfection Indicator (Dharmacon, Lafayette, CO).

### Immunoblot assays

Cultured cells were lysed with ice-cold Pierce RIPA Lysis and Extraction Buffer (Thermo Fisher Scientific, Waltham, MA) with Halt Protease and Phosphatase Inhibitor Cocktail (Thermo Fisher Scientific, Waltham, MA). Proteins were resolved by SDS-PAGE before transfer to Trans-Blot Turbo Mini 0.2 µm PVDF Transfer Packs (Bio Rad, Hercules, CA). Membranes were blocked with 5% nonfat milk in Tris-buffered saline (Bio Rad, Hercules, CA). Tris-buffered saline with 0.1% Tween-20 (TBS-T, Bio Rad, Hercules, CA) incubation for 1 hour was before overnight incubation at 4°C with primary antibody diluted in 1% nonfat milk or 1% BSA in TBS-T. Three 10-minute washes in TBS-T solutions were done with an hour incubation with secondary antibody diluted in 5% nonfat milk. After three additional 10-minute TBS-T washes, visualization was done by Clarity Western ECL substrate (Bio Rad, Hercules, CA) and quantification was by ImageJ software (imagej.nih.gov/ij) and ImageLab (Bio Rad, Hercules, CA). Antibodies were as follows: anti-USP18 (Cell Signaling Technology, Beverly, MA), anti-β-Actin (Cell Signaling Technology, Beverly, MA), anti-β-Actin (Santa Cruz Biotechnology, Dallas, TX), anti-14-3-3ζ (Santa Cruz Biotechnology, Dallas, TX), anti-ISG15 (Cell Signaling Technology, Beverly, MA), anti-IgG (Abcam, Cambridge, United Kingdom), anti-HA tag (Cell Signaling Technology, Beverly, MA), and anti-USP18 (Cell Signaling Technology, Beverly, MA). Antibodies recognizing USP18 were described.^21^ Secondary anti-murine and anti-rabbit antibodies were purchased (Bio Rad, Hercules, CA). Between 50 µg and 100 μg of protein was loaded in each well. Immunoblots were independently performed at least in three separate replicate experiments and a representative immunoblot was displayed. To exclude inter-experimental variation, all three independent immunoblots were quantified, and the averages of all three respective experiments were displayed. Immunoblots were stripped using Restore PLUS Western Blot Stripping Buffer (Thermo Fisher Scientific, Waltham, MA). Stability assays for 14-3-3ζ protein were with cycloheximide (Selleck Chemicals, Houston, TX) treatments (20 μmol/L) for different time periods after lung cancer cell-line transfection, with engineered gain of USP18 expression versus controls.

### Quantitative real-time PCR assays

Real-time PCR (qRT-PCR) assays were performed as before.^21^ Primers were human 14-3-3ζ primer (Hs00237047_m1, Thermo Fisher Scientific, Waltham, MA) and murine 14-3-3ζ primer (Mm01158417_g1, Thermo Fisher Scientific, Waltham, MA); human USP18 primer (Hs00276441_m1, Thermo Fisher Scientific, Waltham, MA) and murine USP18 primer (Mm01188805_m1, Thermo Fisher Scientific, Waltham, MA); human actin primer (Hs01060665_g1, Thermo Fisher Scientific, Waltham, MA); and murine actin primer (Mm01205647_g1, Thermo Fisher Scientific, Waltham, MA).

### Stable transfection

Stable USP18 transfectants were utilized as before.^21^ USP18 protein levels were assessed by immunoblot analyses. Two different shRNAs with the greatest knock-down efficiency were individually used in these experiments versus controls. The assays were in triplicate and with at least three independent biological replicates.

### Proliferation assays

Logarithmically growing cells were at optimized densities for each lung cancer cell line before seeding onto individual wells of 96 well tissue culture plates (for high-throughput testing). The assays were in triplicates and with at least three independent biological replicates. Proliferation was assessed with the In Vitro Toxicology Assay Kit (Sigma-Aldrich, St. Louis, MO).

### Wound-healing assays

Lung cancer cells were seeded at a density of 1 × 10^6^/ml onto individual wells of 24-well tissue culture plates. Sterile pipette tips were used for wound scratch assays and rinsing was done with phosphate buffered saline (PBS) before taking images (10X magnification) at baseline and 24 hours after scratching. The assays were in triplicate and with at least three independent biological replicates.

### Migration and invasion assays

Migration assays were made with Transwell migration chambers (Costar, 8 μm pore size; Sigma-Aldrich, St. Louis, MO) and the vendor’s procedures. For each lung cancer cell line 8 × 10^4^ cells were plated onto individual wells. After 18 hours incubation for human and 24 hours for murine lung cancer cell lines fixation was with 70% methanol, stained with crystal violet and counted using a Nikon Eclipse ts2 microscope.

Invasion assays were with matrigel invasion chambers (Corning, Corning, NY) following the manufacturer’s methods. After 18 hours incubation for human and 24 hours for murine lung cancer cell lines fixation was with 70% methanol, staining was with crystal violet, and counting was with a Nikon ECLIPSE Ts2 microscope. The assays were in triplicate with at least three independent biological replicates.

### Mouse models

The 129S2/SVPasCrl murine line was obtained from Dr. Jonathan Kurie (MD Anderson Cancer Center, Houston, TX). For murine syngeneic metastasis experiments, 1 × 10^6^ 344SQ USP18 stable knock-down or control transfectants were injected into the flanks of male mice to assess metastases, as before.^36^ Each study group had 15 mice. All mice were sacrificed 2 months post-injection using Institutional Animal Care and Use Committee (IACUC)-approved protocols. Lung and primary tumors were harvested, measured, formalin-fixed, and paraffin-embedded. For tail-vein injection metastases experiments, 7 × 10^3^ 344SQ control or USP18 siRNA knock-down cells were injected, respectively, into male mice. Each group had 10 mice. Independent replicate experiments were done. Mice were euthanized at 7 days using IACUC-approved protocols. Lung tissues were harvested, measured, formalin-fixed, and paraffin-embedded. All studies were reviewed and approved by the IACUC at MD Anderson Cancer Center.

### Reverse-phase protein array

The RPPAs were by the Functional Proteomics RPPA Core Facility of MD Anderson Cancer Center as described at https://www.mdanderson.org/research/research-resources/core-facilities/functional-proteomics-rppa-core.html and optimized methods were used.^38^

### Immunoprecipitation

Cells were washed with PBS (Corning, Corning, NY) and lysed using ice-cold Pierce RIPA Lysis and Extraction Buffer (Thermo Fisher Scientific, Waltham, MA) supplemented with Halt Protease and Phosphatase Inhibitor Cocktail (Thermo Fisher Scientific, Waltham, MA). Immunoprecipitation experiments were with anti-HA (BioLegend, San Diego, CA) pull-downs and with Protein A/G PLUS-Agarose (Santa Cruz Biotechnology, Dallas, TX).

### Mass spectrometry

The 293T cells were harvested 48 hours after transfection. Cells were washed twice with PBS buffer before lysis in ice-cold Pierce RIPA Lysis and Extraction Buffer supplemented with protease inhibitor cocktail. Total protein concentration was determined by the Bio-Rad protein assay (Bio Rad, Hercules, CA). Immunoprecipitation was done with SDS-PAGE and Coomassie blue staining. Proteins from 20 to 37 kDa and 37 to 75 kDa were excised from gels for 14-3-3ζ proteomics analysis using detection and quantification as before.^39^

### Immunohistochemistry

De-identified formalin-fixed, paraffin-embedded NSCLC cases with adjacent histopathologically benign lung were displayed on a tissue array obtained from the Division of Cancer Treatment and Diagnosis of the National Cancer Institute (Frederick, MD).^40^ Forty-nine NSCLC cases were examined. Formalin-fixed and paraffin-embedded 344SQ primary lung cancers were harvested with lung tissues harboring metastasis from the murine syngeneic 129S2/SVPasCrl cancer model after independent transplantation of control or stable USP18 knock-down transfectants. Immunohistochemistry for 14-3-3ζ was with a Leica BOND RX automated stainer and Leica Bond Polymer Refine Detection reagents (Leica Microsystems Inc, Buffalo Grove, IL). Antibody for 14-3-3ζ detection was purchased (Thermo Fisher Scientific, Waltham, MA) with recombinant 14-3-3ζ protein (Abcam, Cambridge, MA) for blocking experiments.

Immunohistochemistry for 14-3-3ζ was independently performed in the human normal–malignant lung arrays. The assays were independently performed in murine primary versus metastatic lung cancers following transplantation of USP18 knock-down versus control transfectants of murine syngeneic 344SQ lung cancer cells. This was done to score syngeneic metastasis using an Aperio AT2 scanner (Leica Biosystems, Buffalo Grove, IL) and whole slide digital images.

Analyses of images and data were supported in part by the NCI HALO Image Analysis Resource (Indica Labs, Corrales, NM) and by the Molecular Histopathology Laboratory at Frederick National Laboratory. Slides were annotated by a pathologist who was unaware of the murine treatment arms or of the clinical information for the human histopathologic specimens. Artifact and necrosis within the primary tumor were excluded from analysis. Image analysis was done using the cytonuclear algorithm in HALO version 3.2 to determine percent-positive cells and the H score for 14-3-3ζ within the primary and metastatic lung cancers. Lung lobes were annotated including metastases. Random forest tissue classifier was used to differentiate cancers from the normal lung. Two pathologists independently reviewed and scored the histopathologic findings. Any scoring differences were addressed on reanalysis.

### TCGA analysis

Kaplan–Meier survivals were with the log-rank test and the ‘survival’ package in the R software. For these analyses, high (above median) expression versus low (below median) levels of expression were compared.

## Statistical analysis

Statistical analyses were with SPSS Statistics software (version 23, SPSS) and GraphPad Prism software (version 8, GraphPad Software). Data were mean with Standard Deviations (SDs). Two-tailed Student t tests compared different studied groups. Independent experimental results were pooled for statistical assessments. Experiments were in triplicate with at least three biological replicates except for *in vivo* murine experiments that were two independent replicates.

## Results

### USP18 regulates lung cancer cell growth

**Figure 1.**
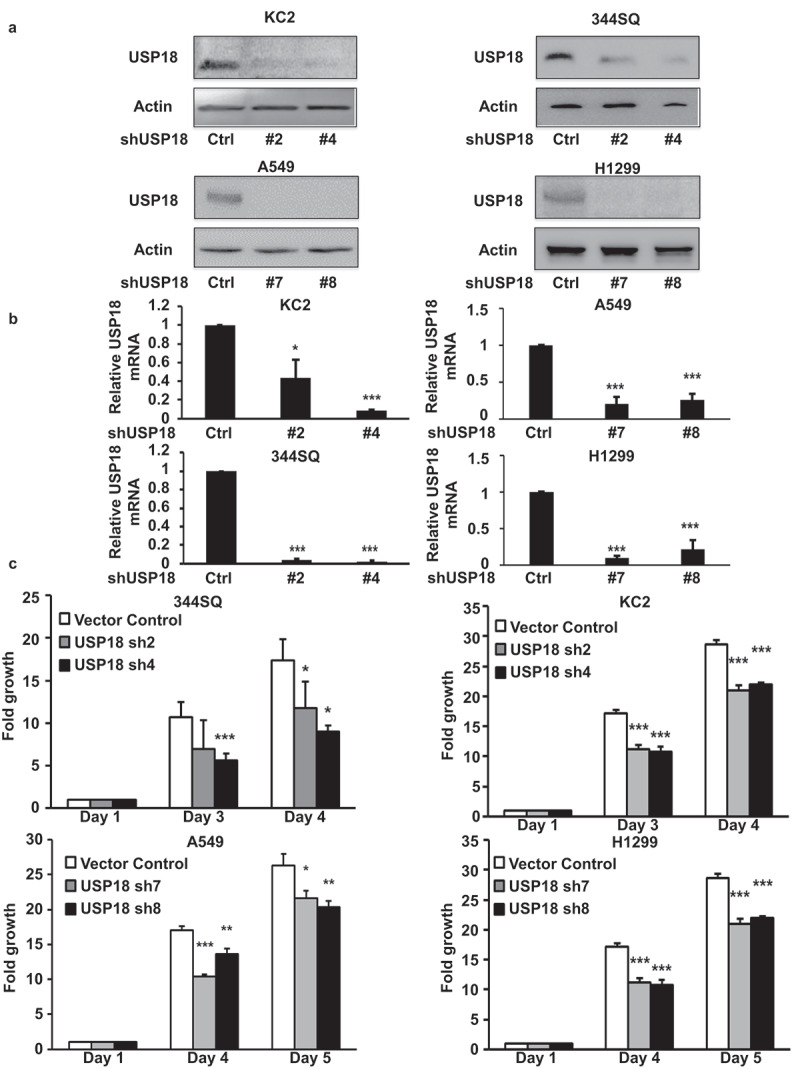
Knock-down of USP18 expression statistically-significantly decreased growth of lung cancer cell lines. (a-b) USP18 knock-down by individual shRNAs was independently achieved in two murine and two human lung cancer cell lines. Immunoblot and real-time qPCR assays were done to validate shRNA knock-down of USP18. (c) Knock-down of USP18 expression inhibited proliferation of human (A549 and H1299) and murine (KC2 and 344SQ) lung cancer cell lines, as measured by proliferation assays that were normalized to control (inactive) shRNA transfected lung cancer cell lines. The symbols refer to * P < .05, ** P < .01, and *** P < .001, respectively.

The DUB USP18 affects protein stability.^21–23^ Loss of USP18 expression reduced lung cancer cell-line growth and lung tumor formation after transfectants were tail-vein injected into syngeneic mice.^21^ To explore comprehensively how USP18 altered the growth of lung cancer cells, engineered USP18 knock-down was achieved independently by shRNAs, respectively, in murine and human lung cancer cell lines versus controls. USP18 knock-down was confirmed by immunoblot and RT-qPCR assays in Figure 1a and 1b. Proliferation assays were performed. Growth of transfectants was statistically significantly decreased in the USP18 knock-down group relative to controls. Kinetic changes appear in Figure 1c.

### USP18 affects lung cancer migration and invasion

**Figure 2.**
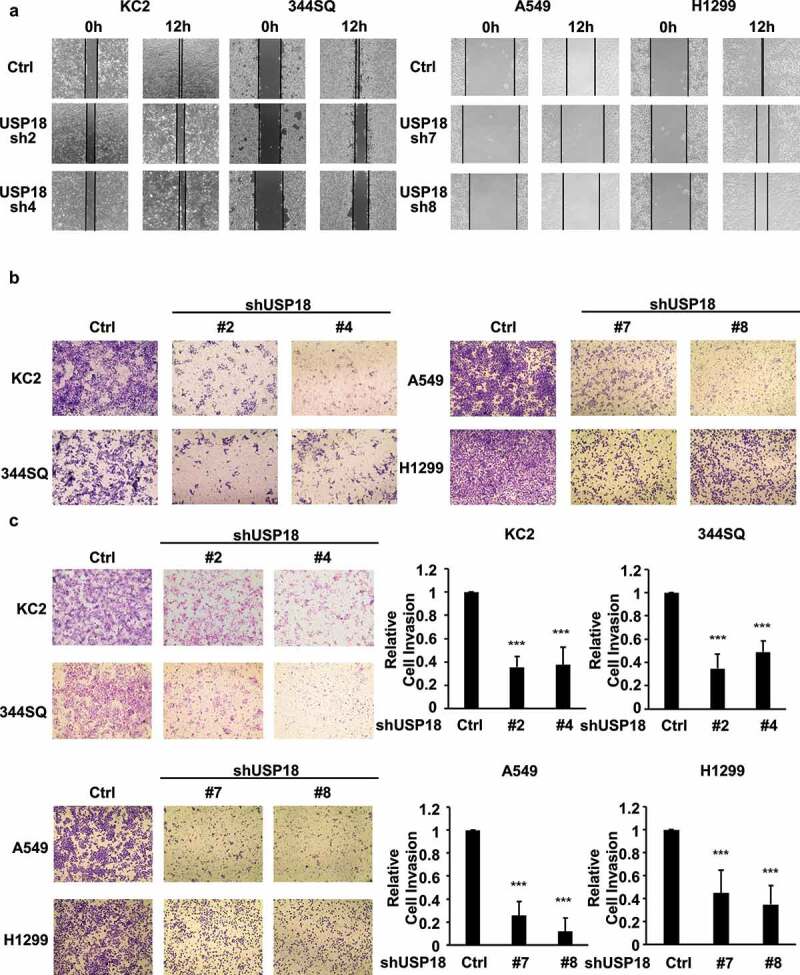
USP18 knock-down led to a statistically significant reduction of migration and invasion of the indicated lung cancer cell lines. (a) Individual USP18 knock-down in transfectants reduced human and murine lung cancer cell migration as measured by wound healing assays that were normalized to control (inactive) shRNA stable lung cancer cell line transfectants. (b) USP18 knock-down of USP18 decreased human and murine lung cancer cell migration as measured by transwell assays that were normalized to control (inactive) shRNA lung cancer cells. (c) Independent knock-down of USP18 expression repressed human and murine lung cancer cell invasion as measured by transwell assays that were normalized to control (inactive) shRNA lung cancer cell line transfectants. The symbol *** refers to P < .001.

To investigate consequences of USP18 knock-down on lung cancer migration, invasion and wound healing-independent assays were performed on stable transfectants using two human and two murine USP18 knock-down lung cancer cell lines. Wound healing and transwell assays showed that USP18 knock-down inhibited these features in the examined lung cancer cell lines at 12 or 24 hours after plating in Figure 2a and 2b. The cell migration ratios of the wound healing assays are presented in **Supplemental Fig. 1**. To learn whether USP18 knock-down affected lung cancer cell invasion matrigel invasion chamber assays were performed independently in two human and two murine lung cancer cell lines having engineered USP18 knock-down versus controls. Findings revealed that the number of cells that transversed the membrane was statistically significantly lower in the USP18 knock-down relative to the control group at 24 hours in Figure 2c. Thus, USP18 knock-down repressed lung cancer cellular invasion.

**Figure 3.**
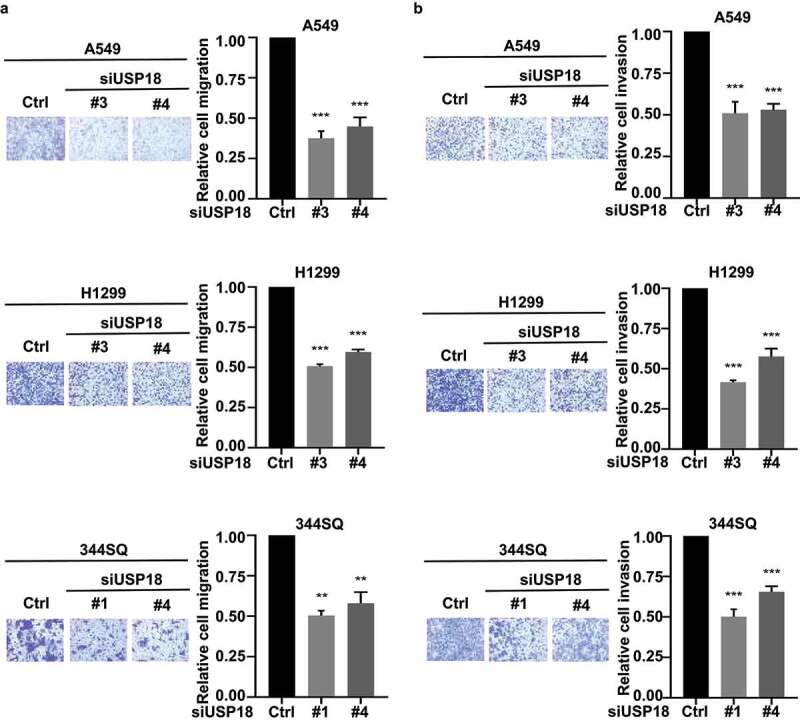
Independent knock-down of USP18 expression by siRNAs led to a statistically significant reduction of migration and invasion in the examined human and murine lung cancer cell lines. (a) Knock-down of USP18 activity reduced human and murine lung cancer cell migration as measured by transwell assays that were normalized to control (inactive) siRNAs transiently transfected lung cancer cell lines. (b) This siRNA-mediated reduction of USP18 activity decreased the invasive ability of human and murine lung cancer cell lines as measured by transwell assays that were normalized to control (inactive) siRNA transfected lung cancer cell lines. The symbols indicate ** P < .01 and *** P < .001, respectively.

Migration and invasion assays were independently performed on these USP18 knock-down lung cancer cell lines after individual introduction of USP18-targeting siRNAs versus control siRNAs. USP18 expression profiles of these different transfectants were assessed by qRT-PCR assays in **Supplemental Fig. 2**. Results independently confirmed similar patterns of repression of migration and invasion as with USP18 shRNA-mediated inactivation in Figure 3a and 3b.

### USP18 controls lung cancer metastasis

**Figure 4.**
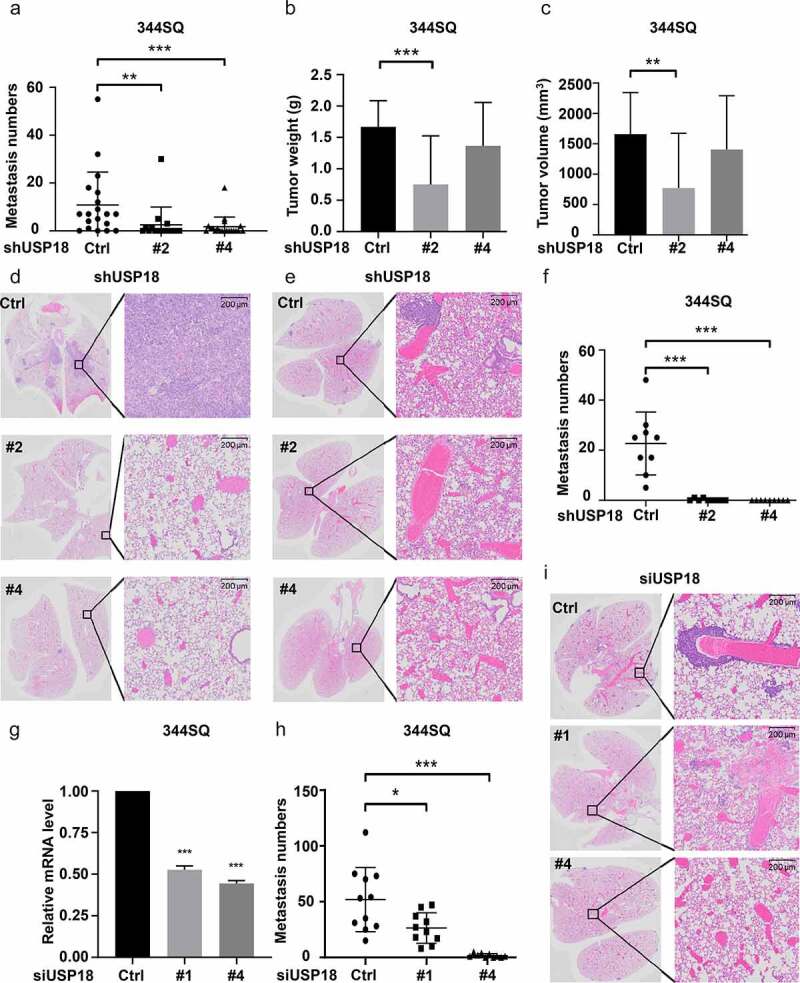
Down-regulation of USP18 expression statistically-significantly reduced lung cancer metastasis. (a-d) USP18 stable knock-down by the indicated shRNAs introduced into murine 344SQ lung cancer cells decreased their metastatic capacity. These findings display: (a) lung metastasis number, (b) primary tumor weight and (c) primary lung tumor volumes. These outcomes were measured 8 weeks after subcutaneous injection and presented as mean ± SEM (N = 15). (d) Representative photomicrographs of lung tissues bearing metastasis from a subcutaneous tumor were stained with hematoxylin (H) and eosin (E) as displayed. USP18 stable knock-down achieved by transfection of the indicated syngeneic murine lung cancer cell line that was transfected with USP18-inactivating versus control shRNAs decreased pulmonary metastasis in this tail-vein injection lung cancer metastasis model. (e) Representative images of lung tissues harboring metastasis from a respective tail vein injection that were stained with H and E are displayed. (f) Lung tumor nodule numbers were measured 9 days after tail-vein injection and are presented as mean ± SEM (N = 10). (g-i) USP18 knock-down by the indicated siRNAs transfected into the syngeneic 344SQ murine lung cancer cell decreased pulmonary metastasis. (g) Real-time qPCR assays were done to validate siRNA knock-down of USP18 expression. (h) Lung nodule numbers were measured 9 days after injection and presented as mean ± SEM (N = 10). (i) Representative photomicrographs of the indicated lung tissues were scored for metastasis by H and E staining. The symbols refer to * P < .05, ** P < .01 and *** P < .001, respectively.

The relationship between USP18 expression and lung cancer metastasis was explored using two syngeneic lung cancer models. The first model used the 344SQ murine lung cancer cell line and previously described protocols.^36^ The indicated lung cancer cell lines were engineered to have USP18 repression using different shRNAs. Findings were compared to control shRNA transfectants. Each transfectant was injected (1 × 10^6^ cells/mouse) into the flank of each mouse with 15 mice independently injected in each group. Lung cancer cells spontaneously metastasized to the lungs in this model within 8 weeks.^36^ Mice were monitored during this study time period (three times a week) and were sacrificed following IACUC-approved methods. The primary flank cancers and lungs from each mouse were harvested; lung cancer numbers of macroscopic metastatic tumors were scored and histopathologically analyzed. Numbers of lung tumors were statistically-significantly lower in the USP18 knock-down group versus controls in Figure 4a. Volumes and weights of the respective primary lung tumors decreased in the USP18 knock-down group versus controls in Figure 4b and 4c. Hematoxylin (H) and eosin (E) staining of these transfected metastatic lung cancers appears in Figure 4d.

The second lung cancer metastasis model used tail vein injection of USP18 knock-down transfectants achieved by independent introduction of different siRNAs into the 344SQ murine lung cancer cell line. Knock-down of USP18 was confirmed by RT-qPCR assays (Figure 4g) with 7 × 10^3^ cells injected into each mouse. Each studied group had 10 mice. Mice were monitored over a nine-day period and humanely sacrificed according to IACUC-approved procedures. Lung tissues were harvested from the respective study groups and analyzed using histopathologic assays. Numbers of visible lung nodules were statistically significantly lower in the USP18 knock-down group versus controls in Figure 4f (shRNAs) and 4h (siRNAs). H and E histopathology images were displayed for these groups in Figure 4e (shRNAs) and 4i (siRNAs). These findings implicated USP18 in regulating lung cancer metastasis. Both subcutaneous injection metastasis and tail-vein injection metastasis models showed comparable results. Each model validated findings from the other.

### USP18 knock-down and 14-3-3ζ repression

**Figure 5.**
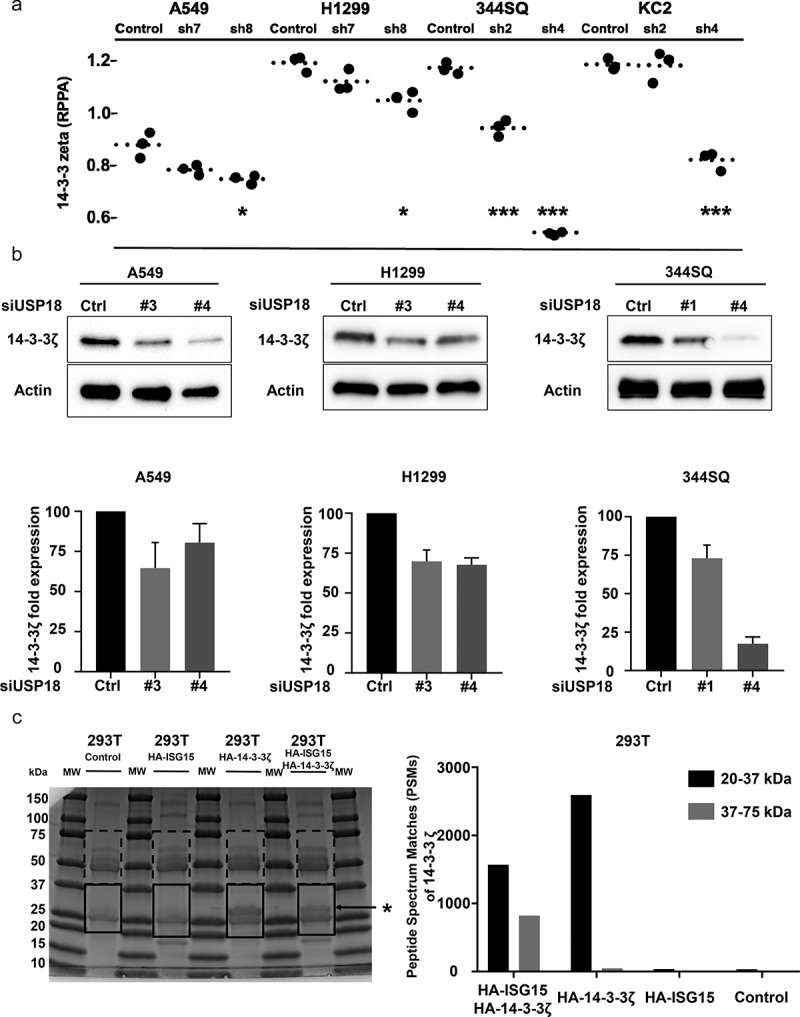
USP18 statistically-significantly affected 14-3-3ζ protein expression in lung cancer cells. (a) Knock-down of USP18 expression by individual shRNAs was independently achieved in two murine and two human cancer cell lines. As displayed in this figure this led to decreased 14-3-3ζ expression as measured by RPPAs. (b) Immunoblot assays were done to validate the results. The symbols indicated *P < .05 and *** P < .001, respectively. (c) HA-pull-down was done independently for control, HA-ISG15, HA-14-3-3ζ and HA-ISG15 + HA-14-3-3ζ co-transfection in 293 T cellular lysates. Proteins were loaded in each lane as shown. Gel regions between 20kDa and 37kDa (boxes with solid lines) and those between 37kDa and 75kDa (boxes with dashed lines) were separately isolated, digested with trypsin and analyzed by mass spectrometry. The asterisk indicated successful pull-down of HA-14-3-3ζ protein. This figure showed in isolated 293 T cellular lysates that human 14-3-3ζ peptides were detected by mass spectrometry.

To explore the engaged mechanisms, RPPA analyses were done to compare protein expression of key growth-regulatory species between lung cancer cells expressing control shRNAs versus those having shRNA-targeting USP18 for repression achieved independently in two human and two murine lung cancer cell lines (**Supplemental Fig. 3**). RPPAs quantified the expression levels of >300 growth-regulatory proteins using validated antibodies for protein detection (primary data are provided). Decreased 14-3-3ζ expression was observed in all of these lung cancer cell lines (Figure 5a). Decreased 14-3-3ζ levels occurred in cells having USP18 knock-down as confirmed by immunoblot assays (Figure 5b).

The 14-3-3 family member 14-3-3ζ functions as an oncogene in cancers and alters cellular proliferation, migration, and invasion.^25–31^ Mass spectroscopy established that ISG15 complexes with 14-3-3ζ protein in Figure 5c. Cycloheximide treatments of USP18 transfected cells stabilized endogenous 14-3-3ζ protein (**Supplemental Figs. 4A** and **4B**). Since 14-3-3ζ protein expression was regulated by altering USP18 activity, it was hypothesized that USP18 regulated lung cancer migration and invasion by controlling 14-3-3ζ expression.

**Figure 6.**
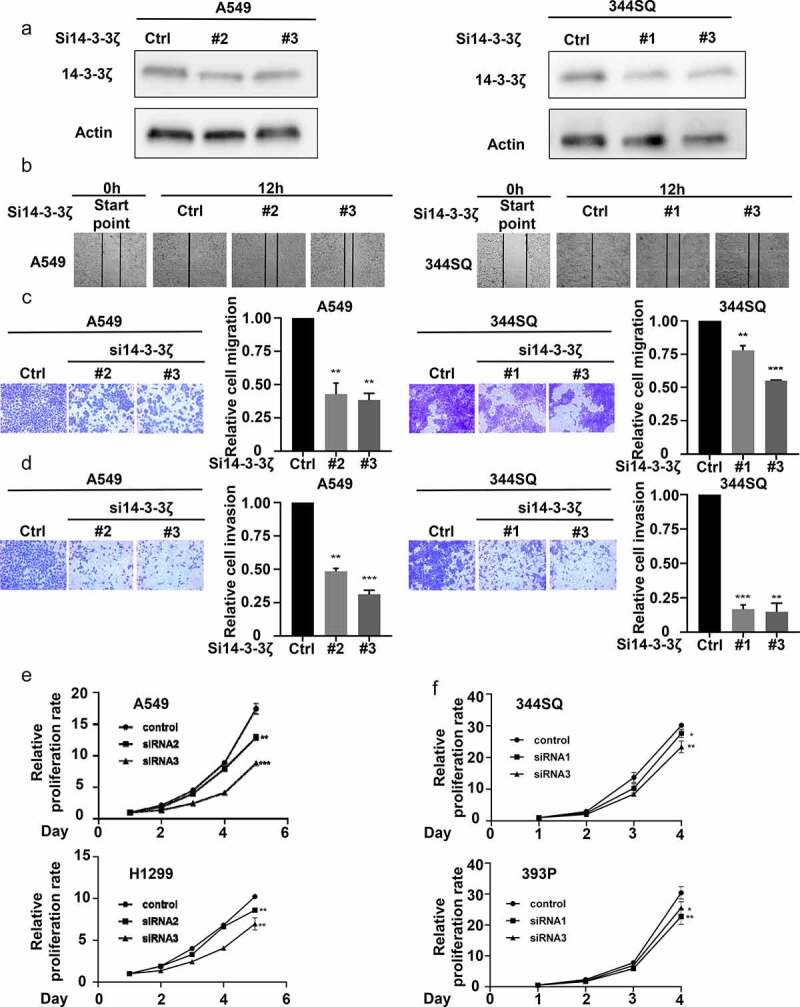
Engineered 14-3-3ζ knock-down led to a statistically significant repression of proliferation, migration and invasion of cultured lung cancer cells. (a) This reduction of 14-3-3ζ expression by individual siRNAs was independently achieved in A549 and 344SQ lung cancer cell transfectants. Immunoblot assays were done to validate the extent of siRNA-mediated knockdown of 14-3-3ζ expression. (b) This 14-3-3ζ knock-down decreased A549 and 344SQ lung cancer cellular migration as measured by wound healing assays that were normalized to control (inactive) siRNA-transfected lung cancer cells. Knock-down of 14-3-3ζ expression reduced A549 and 344SQ lung cancer cell line (c) migration and (d) invasion, as scored by transwell assays that were normalized to control (inactive) siRNA lung cancer cellular transfectants. (e-f) Knock-down of 14-3-3ζ expression inhibited proliferation of human (A549, H1299) and murine (393P, 344SQ) lung cancer cell lines as measured by proliferation assays that were normalized to control (inactive) siRNA lung cancer transfectants. The symbols refer to * P < .05, ** P < .01 and *** P < .001, respectively.

To establish the role of 14-3-3ζ in altering lung cancer cell wound healing and migration, assays were performed after 14-3-3ζ knock-down of lung cancer cells. Transfectants were independently achieved by individual siRNAs and knock-downs were confirmed by immunoblot assay (Figure 6a). Both wound healing and transwell assays showed that 14-3-3ζ knock-down decreased the ability of the examined lung cancer cells to migrate at 12 or 24 hours in the examined lung cancer cell lines (Figure 6b and 6c). To elucidate the consequences of 14-3-3ζ knock-down on lung cancer cell invasion, matrigel invasion chamber assays were performed in lung cancer cells having engineered reduction of 14-3-3ζ expression. The number of engineered lung cancer cells that transitioned across the membrane were statistically significantly lower in the 14-3-3ζ knock-down versus controls scored at 24 hours. This finding revealed that 14-3-3ζ knock-down reduced the invasive ability of lung cancer cells (Figure 6d). Proliferation assays were independently performed after engineered knock-down of 14-3-3ζ in two human and two murine lung cancer cell lines. Lung cancer cell growth significantly decreased in 14-3-3ζ knock-down versus control groups at 72 hours after plating (Figure 6e and 6f). These findings indicated that 14-3-3ζ directly affected lung cancer cell proliferation, migration, and invasion.

### 14-3-3ζ expression and cancer patient outcomes

**Figure 7.**
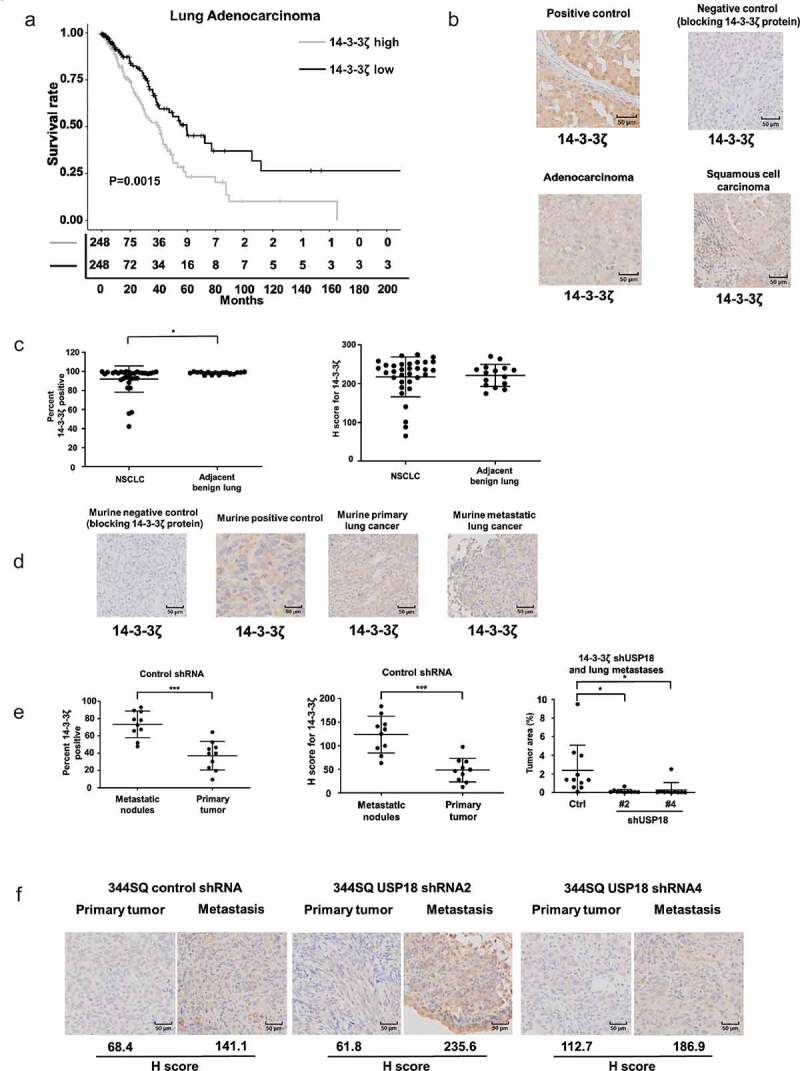
Association between 14-3-3ζ expression and human lung adenocarcinoma survival are presented along with expression profiles for 14-3-3ζ proteins in human and murine lung cancers. Panel A interrogated using TCGA database the Kaplan-Meier analysis of unfavorable survival of human lung adenocarcinomas (P = .0015) that exhibit higher levels of 14-3-3ζ expression versus those exhibiting lower expression. Panel B provides representative immunohistochemical staining images respectively for a 14-3-3ζ positive control (human breast cancer), a negative control with recombinant 14-3-3ζ protein blocking as well as expression profiles for human lung adenocarcinoma and squamous cell carcinoma cases. (c) Immunohistochemical expression of 14-3-3ζ is shown for non-small cell lung cancer (NSCLC) cases versus adjacent histopathologically benign lung with results displayed as the percent expression (left panel) and H-index (right panel). The symbol * refers to a statistical difference (P < .05). (d) This panel shows representative immunohistochemical expression profiles for murine 14-3-3ζ protein with independent blocking protein negative control, positive control, primary murine lung cancer and metastatic lung cancer images. (e) The percent (left panel) and H-index (middle panel) for 14-3-3ζ immunohistochemical expression in primary versus metastatic murine lung cancers showed statistically significant higher levels of 14-3-3ζ in metastatic versus primary lung cancers in shRNA-control transfectants (* P < .05 and *** P < .001). (f) In marked contrast the lung cancers of shRNA-targeting USP18 exhibited a statistically significant decrease in metastatic lesions (right panel). In both shRNA control and shRNA-targeting USP18 cases the H-index of 14-3-3ζ expression was much higher in metastatic murine lung cancers as compared to the paired primary from the same mouse.

Since knock-down of 14-3-3ζ expression inhibited lung cancer cell growth, migration, and invasion, it was examined if enhanced 14-3-3ζ expression in human lung cancers affected survival of lung cancer patients. TCGA analysis revealed that a signature of high 14-3-3ζ expression levels led to a statistically significant and unfavorable survival outcome as compared to lung adenocarcinomas having lower 14-3-3ζ expression profiles (P = .0015, Figure 7a). Immunohistochemical staining is presented in Figure 7b respectively for a 14-3-3ζ positive human breast cancer, a negative control obtained with recombinant 14-3-3ζ protein blocking and expression profiles for lung adenocarcinoma and squamous cell carcinoma cases. Immunohistochemical expression for 14-3-3ζ is shown for non-small cell lung cancer (NSCLC) cases versus adjacent histopathologically benign lung in Figure 7c with results depicted with percent expression (left panel) and H-index (right panel). Varying levels of 14-3-3ζ immunohistochemical expression were detected in The Human Protein Atlas (**Supplemental Fig. 5A**). This was detected across different NSCLC subtypes and stages (**Supplemental Fig. 5A** and **5B)** as scored by the percent and H-index expression profiles for 14-3-3ζ.

Representative immunohistochemical expression profiles for murine 14-3-3ζ expression was assessed. Independent blocking protein-negative controls were interrogated along with positive controls. Primary murine lung cancer and metastatic lung cancer images appear in Figure 7d along with the percent (left panel) and H-index (middle panel) for 14-3-3ζ immunohistochemical expression in primary versus metastatic murine lung cancers. Notably, metastatic murine lung cancers as compared to the corresponding primary lung cancers showed statistically-significantly higher levels of 14-3-3ζ expression. Lung tissues of shRNA-targeting USP18 exhibited a marked and statistically significant decrease in metastases (Figure 7e right panel). In shRNA control and shRNA-targeting USP18 cases, the H-index of 14-3-3ζ expression was much higher in metastatic murine lung cancers as compared to the paired primary from the same mouse in Figure 7f.

To learn if similar survival outcomes occurred in other human malignancies TCGA studies assessed the clinical consequences of high versus low 14-3-3ζ expression profiles in different cancers. A signature of high 14-3-3ζ expression conferred an unfavorable survival outcome (as compared to all other examined cases with the same histopathologic diagnoses) for pancreatic adenocarcinoma (P = .0022, **Supplemental Fig. 6A**), breast cancer (P = .0025, **Supplemental Fig. 6B**), kidney chromophores (P = .028, **Supplemental Fig. 6C**), renal papillary cell carcinoma (P = .032, **Supplemental Fig. 6D**) and uveal melanoma (P = .0074, **Supplemental Fig. 6E**).

### 14-3-3ζ rescue experiments

**Figure 8.**
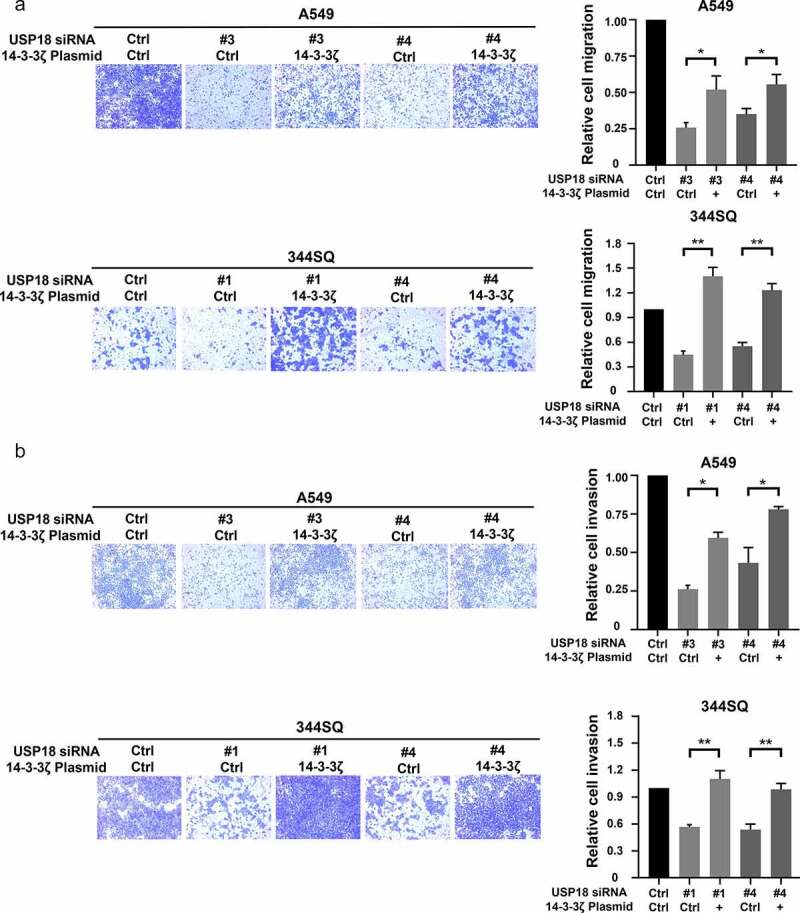
Gain of 14-3-3ζ expression rescued reduction of migration and invasion by siRNA-mediated USP18 knock-down in lung cancer cells. (a) Gain of 14-3-3ζ expression rescued migration in human A549 and murine 344SQ USP18 knock-down lung cancer cell lines as scored by transwell assays normalized to empty vector controls. (b) Gain of 14-3-3ζ expression antagonized the reduction of invasive potential of human A549 and murine 344SQ lung cancer cell lines that had USP18 knock-down measured by transwell assays normalized to control transfectants. The symbols refer to * P < .05 and ** P < .01 , respectively.

Given that 14-3-3ζ levels were associated with USP18 expression, it was explored if USP18 expression affected lung cancer migration and invasion following induced changes in 14-3-3ζ expression. Engineered over-expression of 14-3-3ζ protein was confirmed by immunoblot assays in **Supplemental Fig. 7**. Migration and invasion assays were performed with lung cancer cells independently transfected with USP18-targeting siRNAs or control siRNAs along with transfection of a 14-3-3ζ expression vector or a control vector. Numbers of cells that crossed the membranes were statistically-significantly lower in the USP18 knock-down versus the control groups in these assays. The number of cells that crossed the membrane were statistically significantly rescued when 14-3-3ζ was over-expressed versus control transfectants as shown in Figure 8a and 8b. Engineered reduced USP18 expression in the indicated lung cancer cell lines inhibited the ability of lung cancer cells to migrate and invade. Thus, consequences of USP18 repression were rescued by 14-3-3ζ.

## Discussion

Lung cancer is the most common cause of cancer death; metastasis alters lung cancer morbidity and mortality.^1,4^ A better understanding of cancer metastasis could improve therapy for lung and other human cancers. This study advanced prior work by demonstrating a direct link exists between expression of USP18 and 14-3-3ζ proteins. The USP18 enzyme was shown here to regulate 14-3-3ζ protein levels likely through ISG15ylation. Notably, 14-3-3ζ knock-down markedly decreased lung cancer metastasis. These findings underscore the anti-neoplastic consequence of antagonizing USP18 activity.

As a DUB family member, USP18 removes ISG15 from complexed proteins to alter their stability.^13^ Prior work found 158 proteins coupled to ISG15.^35^ These species are involved in DNA cleavage, chromatin remodeling, polymerase II transcription, growth, metabolism, interferon signaling, development, protein expression, and other USP18-associated activities.^35^ USP18 stabilizes targeted proteins through its DUB enzymatic activity. This affects the actions of oncogenic and tumor suppressive proteins.^10^ Direct evidence for ISG15 regulating 14-3-3ζ protein was shown here using mass spectroscopy that confirmed ISG15 complex formation with 14-3-3ζ protein. This affected 14-3-3ζ protein stability. These new 14-3-3ζ protein functions are part of a previously unrecognized USP18-dependent pathway summarized in **Supplemental Fig. 8**.

This study found that engineered loss of USP18 expression markedly reduced the ability of lung cancer cells to migrate and invade. RPPAs revealed that USP18 expression affected 14-3-3ζ protein levels in murine and human lung cancer cells. Several reports implicated enhanced 14-3-3ζ expression in promoting lung cancer metastasis by regulating Par3, TGF-β, or Snail protein expression.^28,29,41^ ISG15 complex formation regulated expression of a different 14-3-3 family member, 14-3-3σ.^41^ Gain of USP18 expression opposed ISGl5ylation and stabilized 14-3-3σ expression.^41^

The 14-3-3 protein family has seven isoforms (β, γ, ζ, σ, ε, η, and τ) having similar structures and sequence similarity.^42^ To explore how the USP18 enzyme affected lung cancer metastasis, this study established that 14-3-3ζ protein underwent ISG15ylation. This regulated USP18 expression and controlled metastasis.

Prior work found that enhanced 14-3-3ζ expression promoted growth, proliferation, metastasis, and inhibited apoptosis in different cancers.^33,42–46^ TCGA data using different statistical methods as reported here revealed that high 14-3-3ζ levels in human malignancies affected overall survival of lung and other human cancers.^47^ The 14-3-3ζ immunohistochemical profiles detected here in malignant versus benign lung were similar to that reported using The Human Protein Atlas (**Supplemental Fig. 5A)**. Some different 14-3-3ζ patterns of expression levels in lung cancers were reported by others^28,29^ and this is likely related to the scoring systems and antibodies used for 14-3-3ζ protein detection. A notable finding of this study was the substantially increased 14-3-3ζ immunohistochemical expression in murine metastatic versus primary lung cancers. That abundant expression was not overcome by knock-down of 14-3-3ζ expression in primary lung cancers, indicating that restoration of 14-3-3ζ expression can maintain migration, invasion, and metastatic effects in lung and other malignancies.

Experiments reported here uncovered an intriguing consequence of USP18 enzymatic activity affecting the biology of lung and other cancers. This was to reduce metastasis by altering 14-3-3ζ expression. USP18 knock-down significantly inhibited the lung cancer metastasis cascade. *In vivo* effects of USP18 on metastases were elucidated using two different murine syngeneic lung metastases models, a tail-vein injection model, and a subcutaneous injection model. In both models, lung metastases after injection into recipient mice of USP18-knocked-down lung cancer cells repressed USP18 expression and metastases versus controls. Results were independently validated in each of these murine lung cancer metastasis models. Notably, 14-3-3ζ expression was markedly higher in the murine metastatic versus primary lung cancers, indicating the importance of 14-3-3ζ expression profiles in the biology of lung cancer metastases.

The relationship between USP18 and 14-3-3ζ protein expression was comprehensively examined. The 14-3-3 protein family regulates critical growth-regulatory pathways.^25^ This family has distinct subtypes; 14-3-3ζ protein has oncogenic effects in breast, lung cancer, liver, and pancreatic cancers.^25,43–46^ Post-translational modifications affect 14-3-3ζ protein. These include changes in phosphorylation, acetylation, methylation, and ubiquitination.^45,46,48^ Phosphorylated and acetylated modifications of 14-3-3ζ protein follow Akt-mediated phosphorylation of the S58 residue of 14-3-3ζ, promoting apoptosis.^48^ HDAC6 acetylated the K49 and K120 sites of 14-3-3ζ and antagonized interactions of Bad and AS160 with 14-3-3ζ, decreasing cell survival.^49^

This study built on prior work and found that 14-3-3ζ protein undergoes ISG15ylation. This alters 14-3-3ζ protein expression and function. A direct link was found between USP18 and lung cancer metastasis. Consistent with this finding was evidence that the DUB USP4 promoted epithelial–mesenchymal transition (EMT) by stabilizing the PRL-3 expression.^50^ USP18 enzymatically removes ISG15 from complexed proteins.^51^ USP18 was previously found to play an oncogenic role in the survival and growth of lung cancer cells.^17–19,21,22^ It was also shown that USP18 enhanced EMT by stabilizing Twist1 expression in glioblastoma.^23^ The present work found that USP18 knock-down reduced 14-3-3ζ expression and inhibited lung cancer metastasis. Likewise, 14-3-3ζ expression altered survival in human lung cancers and other tumors.

In summary, this study discovered a novel consequence of decreased USP18 activity in lung cancer. This was that 14-3-3ζ protein repression reduced cancer invasion, migration, and metastasis. These and other findings presented here provide a strong rationale for the development of a USP18 inhibitor. We propose that such an inhibitor would exert anti-neoplastic effects alone or as part of an optimal combination regimen. This work has implications for the treatment of metastases in lung and other human malignancies. It has translational relevance since combating metastasis is a major unmet need in oncology.

## Supplementary Material

Supplemental Material
